# Smoking cessation in severe mental ill health: what works? an updated systematic review and meta-analysis

**DOI:** 10.1186/s12888-017-1419-7

**Published:** 2017-07-14

**Authors:** Emily Peckham, Sally Brabyn, Liz Cook, Garry Tew, Simon Gilbody

**Affiliations:** 1 Department of Health Sciences, University of York, Heslington, YO10 5DD UK; 2 Department of Sport, Exercise and Rehabilitation, Northumbria University, Newcastle upon Tyne, NE1 8ST UK

**Keywords:** Severe mental ill health, Smoking cessation, Nicotine replacement therapy, Varenicline, Behavioural intervention, Bupropion

## Abstract

**Background:**

People with severe mental ill health are more likely to smoke than those in the general population. It is therefore important that effective smoking cessation strategies are used to help people with severe mental ill health to stop smoking.﻿ This study aims to assess the effectiveness and cost –effectiveness of smoking cessation and reduction strategies in adults with severe mental ill health in both inpatient and outpatient settings.

**Methods:**

This is an update of a previous systematic review. Electronic databases were searched during September 2016 for randomised controlled trials comparing smoking cessation interventions to each other, usual care, or placebo. Data was extracted on biochemically-verified, self-reported smoking cessation (primary outcome), as well as on smoking reduction, body weight, psychiatric symptom, and adverse events (secondary outcomes).

**Results:**

We included 26 trials of pharmacological and/or behavioural interventions. Eight trials comparing bupropion to placebo were pooled showing that bupropion improved quit rates significantly in the medium and long term but not the short term (short term RR = 6.42 95% CI 0.82–50.07; medium term RR = 2.93 95% CI 1.61–5.34; long term RR = 3.04 95% CI 1.10–8.42). Five trials comparing varenicline to placebo showed that that the addition of varenicline improved quit rates significantly in the medium term (RR = 4.13 95% CI 1.36–12.53). The results from five trials of specialised smoking cessation programmes were pooled and showed no evidence of benefit in the medium (RR = 1.32 95% CI 0.85–2.06) or long term (RR = 1.33 95% CI 0.85–2.08). There was insufficient data to allowing pooling for all time points for varenicline and trials of specialist smoking cessation programmes. Trials suggest few adverse events although safety data were not always reported. Only one pilot study reported cost effectiveness data.

**Conclusions:**

Bupropion and varenicline, which have been shown to be effective in the general population, also work for people with severe mental ill health and their use in patients with stable psychiatric conditions. Despite good evidence for the effectiveness of smoking cessation interventions for people with severe mental ill health, the percentage of people with severe mental ill health who smoke remains higher than that for the general population.

**Electronic supplementary material:**

The online version of this article (doi:10.1186/s12888-017-1419-7) contains supplementary material, which is available to authorized users.

## Background

The physical health of people with severe mental ill health (SMI) is poor, with people with a diagnosis of SMI dying 20–25 years earlier than those in the general population [1]. Smoking is one of the most important modifiable risk factors that contributes to this excess mortality [2]. People with SMI tend to smoke more heavily and extract more nicotine from cigarettes than smokers without mental health problems [3], and up to 70% of people with SMI smoke [4].

Whilst the percentage of people who smoke in the general population has been steadily declining, the percentage of people with SMI who smoke has not seen a similar decline [5]. Despite this, when questioned, the percentage of people with SMI who are interested in cutting down or quitting smoking is similar to that of the general population [6]. In 2010 a systematic review was conducted to establish the clinical and cost effectiveness of smoking cessation and reduction strategies for people with SMI to determine the most successful strategies such as the use of pharmacotherapy (e.g. nicotine replacement therapy, varenicline, bupropion) or behavioural interventions [7]. In the United Kingdom, following the publication of guidance issued by the National Institute of Health and Care Excellence (NICE) Guidance PH 48 in 2013 [8], a number of mental health trusts have decided to go smoke free and encourage people with SMI to give up or cut down on their smoking. We have therefore decided to update the 2010 review with the additional inclusion of e-cigarettes as a smoking cessation strategy to provide up to date information on the most effective and cost-effective strategies to help people with SMI cut down or quit smoking.

## Objectives

To assess the effectiveness and cost-effectiveness of smoking cessation and reduction strategies in adults with severe mental ill health.

## Methods

### Search strategy

The protocol for this review has been registered on the PROSPERO register of systematic reviews (http://www.crd.york.ac.uk/PROSPERO/display_record.asp?ID=CRD42015029455).

An electronic search strategy based on that used in our previous review, combining search terms for severe mental ill health, smoking cessation and randomised controlled trials, adapted from terms developed by the Cochrane groups for schizophrenia and tobacco addiction was used to search the following database for potentially relevant studies: MEDLINE (PubMed), EMBASE, PsycINFO, CINAHL, Health Management Information Consortium (HMIC) and CENTRAL.

The search strategy was limited to the inception year of each database until September 2016. An example of the search strategy is shown in an additional word file (see Additional file 1).

Searching other resources.

Reference lists of all identified studies and existing reviews were checked for additional potentially relevant studies.

### Inclusion criteria

#### Types of studies

Randomised controlled trials (RCTs), including cluster-randomised controlled trials, that assess the effects of smoking cessation and reduction interventions in people with severe mental ill health were included. Studies conducted in any country and in either in-patient or out patient settings were eligible for inclusion. Studies that are not published in English were excluded.

#### Types of participants

Participants were adults aged 18 years and above who had been diagnosed with SMI. We defined SMI as schizophrenia or other psychotic disorders, bipolar disorder and depression with psychotic features. We have not included personality disorder, severe anxiety disorder, post traumatic stress disorder (PTSD), major depression or autism in this review. We have based this classification on diagnoses that would typically be included on a UK primary care SMI register [9]. Diagnosis needed to be made by using International Classification of Disease (ICD10 F20–29 and F30–31) or Diagnostic and Statistical Manual (DSM IV 295.x, 296.x and 297.x) criteria.

Studies involving participants who had a problem with substance abuse (other than nicotine addiction) without any other mental disorder, or whose participants had learning disability, dementia, other neurocognitive disorders or terminal illness were not included in this review.

#### Types of interventions

Trials of all types of smoking cessation and reduction strategies, (behavioural or pharmacological as monotherapy or in combination) compared to each other, placebo, usual care or to no intervention were included, including trials of very brief advice. Behavioural interventions include on-to-one programmes, group programmes, and telephone counselling. Pharmacotherapy includes products licensed for smoking cessation e.g. nicotine replacement therapy (NRT), varenicline, nortriptyline, and bupropion. Trials in which electronic cigarettes (‘e-cigarettes’) have been used as a smoking cessation aid were also included. Studies looking at ‘implementation of a smoke-free environment’ as an intervention were excluded. Behavioral interventions were classed as ‘group’ or ‘individual’ therapy.

#### Types of outcome measure

The primary outcome measure was biochemically verified self-reported smoking cessation. Accepted methods of biochemical verification were expired carbon monoxide (CO level of <10 ppm (p.p.m.), salivary cotinine <15 ng/ml, urinary cotinine <50 ng/ml or serum cotinine <15 ng/ml. All follow-up times were included and categorised as short-term quit if less than or up to four weeks, mid term quit for up to six months, and long-term quit if longer than six months. Participants lost to follow up were treated as ‘still smokers’.

The secondary outcomes were:

1. Smoking reduction; as no acceptable standard exists for its measurement, any measure was acceptable as long as it was verified by biochemical assay
2. Change in body weight
3. Change in psychiatric symptoms (any validated symptom scale)
4. Adverse events

#### Selection of included studies and data extraction

Two authors independently screened 10% of the titles and abstracts of publications identified by the search strategy. Results from this initial screening were compared to check the level of agreement between the two authors over which studies should proceed to full text screening. Both authors were in agreement over which texts should proceed to full text screening therefore one author continued to screen the remaining studies. All studies that were not applicable according to our inclusion criteria were discarded. The full text of the remaining references was obtained.

Two authors independently decided whether the studies meet the inclusion criteria with any disagreements resolved through discussion with a third author.

#### Data extraction

Two authors independently extracted data from the included studies. Any disagreements were resolved through discussion with a third author where necessary.

Any missing data, relating to the primary outcome only, was sought by contacting the Investigators and/or corresponding authors of primary studies.

#### Assessment of risk of bias in included studies

The methodological quality of included trials was assessed independently by two reviewers using the Cochrane’s tool for assessing risk of bias, [10] which assesses the following domains:

1. Sequence generation (selection bias)
2. Allocation concealment (selection bias)
3. Blinding of participants and personnel (performance bias)
4. Blinding of outcome assessment (detection bias);
5. Incomplete outcome data (attrition bias)
6. Selective outcome reporting (reporting bias)
7. Other potential sources of bias

Each of the domains was scored as ‘high’, ‘low’ or ‘unclear’ risk of bias, following criteria outlined in Chapter 8 of the Cochrane Handbook for Systematic Reviews of Interventions [10].

#### Data synthesis

A narrative overview of study design features, study populations, outcomes, risk of bias and study results is given.

For smoking cessation data, we present risk ratios with 95% confidence intervals as per our previous review [7]. Where interventions and comparisons were sufficiently similar we conducted a meta-analysis using RevMan (version 5.3, *Review Manager (RevMan) [Computer program]. Version 5.3. Copenhagen: The Nordic Cochrane Centre, The Cochrane Collaboration, 2014*). We performed standard pairwise meta-analysis for every comparison that contained at least two studies and used a random-effects model if studies were statistically heterogeneous as measured by I^2^ (I^2^ ≥ 50%); otherwise we used a fixed-effect model. Absolute quit rate was taken as the proportion of participants who met criteria for abstinence out of the number randomised to that group.

#### Unit of analysis issues

The unit of analysis was the individual.

## Results

**Fig. 1:**
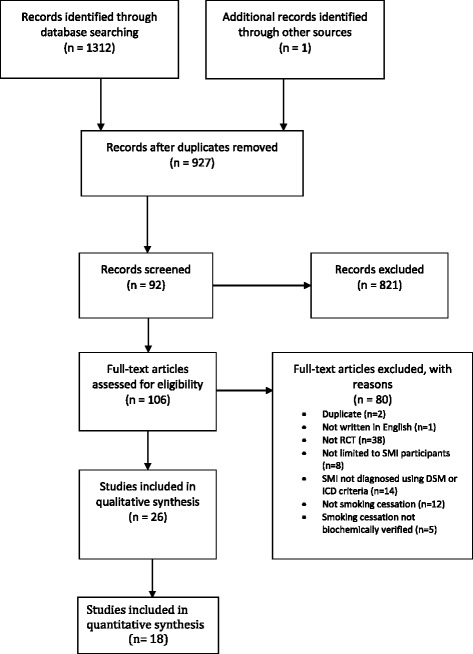
Prisma diagram

Of the 1312 records identified 106 full texts were screened (Fig. 1). Of these 28 (based on 26 studies) involving 1978 participants met the inclusion criteria [11–38]; 18 more studies than in our previous review. The reasons for ineligibility are shown in Fig. 1, with the most common reason being that the study was not a randomised controlled trial.

### Study characteristics

Study characteristics are given in Table 1. No cluster RCTs were identified in this review. The sample size of the studies ranged from five participants [22, 37] to 298 participants [18]. The majority of the studies recruited participants who were outpatients (*n* = 20), one study recruited solely from an inpatient setting [29], and one study recruited from a mixture of inpatients and outpatients [35] the remaining 4 studies did not clearly state whether the participants were inpatients or outpatients.

**Table 1:** Study characteristics

Study/design	Population	Interventions	Smoking abstinence outcomes	Secondary outcomes
Complex interventions				
Baker 2006 [18, 38] (including data from Baker 2010) RCT	298 clinically stable adult outpatients with ICD diagnosis of psychotic disorder who expressed an interest in quitting smoking and smoke ≥15 cigarettes per day. Australia 52% male, ethnicity not stated.	1. Individual motivational interviewing/CBT 2. Usual care Intervention consisted of 8 × 1 hour sessions of manualised motivational interviewing and CBT over 10 weeks.	Continuous abstinence self report verified by expired CO < 10 ppm at 3,6, 12 months and 4 years 7 day point prevalence smoking abstinence verified by expired CO <10 ppm at 3, 6 12 months and 4 years	Change in psychiatric symptoms (BDI, BPRS, SF-12, STAI)
Baker 2015 [32] RCT	235 adult outpatients who expressed an interest in quitting smoking with ICD diagnosis of psychotic disorder and Smoking ≥15 cigarettes per day and with stable symptoms. Australia 59% male, 84% Australian born.	1. Healthy lifestyle intervention (individual) 2. Telephone intervention Healthy lifestyle intervention consisted of manualised motivational interviewing and CBT delivered as a single 90 min sessions followed by 7 × 1 h sessions weekly then 3 fortnightly 1 h sessions then monthly 1 hour sessions for 6 months. The telephone intervention consisted of 1 face to face meeting followed by up to 16 × 10 minute manualised telephone sessions	7 day point prevalence smoking abstinence verified by expired CO <10 ppm at 15 weeks and 12 months verified by expired CO measure Number of cigarettes per day FTND	Change in psychiatric symptoms (BBRS-24, BDI, SF-12 mental component)
George 2000 [12] RCT	45 participants with DSM IV schizophrenia or schizoaffective disorder with a FTND score of ≥5 United States 67% male, 62% white.	1. ALA group programme + NRT patch 2. Specialised group programme + NRT patch *21 mg for 6 weeks then 14 mg for 2 weeks then 7md for 2 weeks ALA group consisted of 3 weekly 60 min manualised sessions of group counselling Specialised programme consisted of 3 weeks of 1 h motivational enhancement then 7 weeks 1 h of psychoeducation. All manualised	7 day point prevalence abstinence at week 10, and 26 verified by expired CO <10 ppm. Continuous abstinence in last 4 weeks of treatment	Change in psychiatric symptoms (AIMS, BDI, PANSS, WEPS)
Gilbody 2015 [33] RCT	97 adult outpatients with DSM IV schizophrenia, schizoaffective disorder or bipolar disorder who expressed a desire to cut down or quit smoking and smoked ≥10 cigarettes per day. England 60% male, 87% white.	1. Bespoke intervention 2. Usual care Intervention consisted of 8-10 × 30 min maunalised sessions tailored to the participants needs.	Smoking cessation at 12 months (CO ≤ 10 ppm) FTND Number of cigarettes per day	Change in psychiatric symptoms (SF-12, PHQ-9)
Smith 2015 [34] RCT	33 outpatients with DSM IV schizophrenia or schizoaffective disorder 73% male, 30% white.	1. 5 sessions of transcranial direct current stimulation 2. 5 sessions of sham treatment	Self report number of cigarettes smoked and expired CO I week after final treatment session Urges to smoke	PANSS and PSYCHRATS hallucination scale
Steinberg 2003 [15] RCT	78 outpatients with DSM IV schizophrenia or schizoaffective disorder smoking ≥10 cigarettes per day United States 68% male, 77% white.	1. Motivational interviewing (individual) 2. Psychoeducational intervention (individual) 3. Control Motivational interviewing consisted of 1 × 40 minute session. Psychoeducation consisted of 1 × 40 minute session Control consisted of 1 5 min session.	Expired CO at 1 week and 1 month Number of cigarettes per day Heaviness of smoking Contemplation ladder FTND Importance of quitting Confidence in ability to quit	
Steinberg 2016 [36] RCT	98 outpatients with DSM IV schizophrenia, schizoaffective disorder or Bipolar I 46% male, 61% white.	1. Motivation interviewing 1 × 45 min personalised session 2. Interactive education 1 × 45 min non personalised session Motivational interviewing 1 45 min session manualised. Interactive education consisted of 1 × 45 min manualised session	Expired CO at 1 month Motivation to quit	
Williams 2010 [23] RCT	100 adult outpatients with DSM IV schizophrenia or schizoaffective disorder who Smoke ≥10 cigarettes per day and were willing to try and quit smoking. United States 64% male, 66% white.	1. Treatment of nicotine addiction in schizophrenia + nicotine patch (individual) 2. Medication management + nicotine patch 3. (individual) *21 mg for 12 weeks and 14 mg for 4 weeks TANS consisted of 24 × 45 min sessions over 26 weeks of manualised motivational interviewing. MM consisted of 9 × 20 min sessions of manualised active education.	7 day point prevalence abstinence at 3, 6 and 12 months verified by expired CO <10 ppm. Continuous abstinence at 3 months.	Change in psychiatric symptoms (BDI, PANSS)
Wing 2012 [28] RCT	15 DSM-IV schizophrenia or schizoaffective disorder, smoking ≥10 cigarettes per day for 3 years or more with expired CO ≥ 10 ppm and FTND score ≥ 4 and motivated to quit within the next month. Ethnicity and gender not reported.	1. Trans cranial magnetic stimulation + weekly group therapy and nicotine patch (21 mg) 2. Sham + weekly group therapy and nicotine patch (21 mg)	Weekly (for 10 weeks) Smoking self report verified by expired CO. Tiffany questionnaire for smoking urges	Change in psychiatric symptoms (PANSS) Adverse events
Bupropion studies				
Evins 2001 [13, 16] (including data from Evins 2004) RCT	19 DSM IV schizophrenia outpatients on a stable dose of antipsychotic medication for at least 4 weeks who smoke at least half a pack of cigarettes per day and express a wish to quit smoking United States 61% male, 89% white.	1. Bupropion (150 mg per day) + CBT Quit Smoking Group 2. Placebo + CBT Quit Smoking group	7 day point prevalence abstinence verified by expired CO < 9 ppm or serum cotinine <14 ng/ml at 12 and 24 weeks and 2 years Significant smoking reduction at12, 24 weeks and 2 years defined by ≥30% reduction in expired CO and ≥50% reduction in number of cigarettes per day	Change in psychiatric symptoms (BPRS, SANS, HamD, AIMS, Hillside Akathisia Scale, SAS)
Evins 2005 [17] RCT	19 DSM-IV schizophrenia or schizoaffective disorder outpatients and smokes 10 cigarettes per day with stable symptoms and on a stable dose of antipsychotic for >30 days HAM-D score ≤ 20 and willing to set a quit date within 4 weeks. United states 68% male, ethnicity not reported.	1. Bupropion (150 mg) + behavioural therapy intervention 2. Placebo + behavioural therapy intervention	7 day point prevalence abstinence at week and week 4, 12 and 24 verified by expired CO <9 ppm. 4 week continuous abstinence at week 24 Number of cigarettes smoked per day	Change in psychiatric symptoms (SANS, Ham-D, Ham-A, PANSSS, SAS, Barnes akathisia scale) Adverse events
Evins 2007 [19] RCT	51 adult outpatients DSM-IV Schizophrenia, capacity to consent, smokes 10 cigarettes per day with stable symptoms and on a stable dose of antipsychotic for 30 days and willing to set a quit date within 4 weeks United States 57% male, ethnicity not reported.	1. Bupropion (150 mg 1 x daily 7 days then 150 mg 2× daily thereafter) + transdermal nicotine patch, nicotine polacrilex gum and CBT 2. Placebo + transdermal nicotine patch, nicotine polacrilex gum and CBT 21 mg/d 4 weeks, 21 mg/d 2 weeks then 7 mg/d 2 weeks 2 mg as needed up to 18 mg/d	7 day point prevalence abstinence at week12, 24 and 52 verified by expired CO <8 ppm. 4 week continuous abstinence at week 8, 12, 24 and 52.	Change in psychiatric symptoms (SANS, Ham-D, STAI, PANSSS)
Fatemi 2013 [30] RCT	24 clinically stable DSM-IV schizophrenia or schizoaffective disorder, smoking ≥10 cigarettes per day expressing a motivation to quit or reduce smoking. United States Ethnicity and gender not reported.	1. Bupropion + antismoking counselling 2. Varenicline + antismoking counselling 3. Placebo + antismoking counselling	Self report abstinence verified by CO Serum and urine levels of nicotine and cotinine	Change in psychiatric symptoms (BPRS, SAPS, SANS, BDI, CSSRS, WISDM, MNWS) Adverse events
George 2002 [14] RCT	32 clinically stable adult outpatients on a stable dose of medication with DSM IV schizophrenia or schizoaffective disorder smoking ≥10 cigarettes per day with expired CO > 10 ppm, plasma cotinine >150 ng/ml and scored ≥5 on FTND and ≥3 on an assessment measure of self-reported motivation indicating a strong desire to quit smoking. US 56% male, 63% white.	1. Bupropion (150 mg 2× day) + specialised schizophrenia smoking cessation program 2.Placebo +specialised schizophrenia smoking cessation program	7 day point prevalence abstinence at week 10, and 36 verified by expired CO <10 ppm. Tiffany questionnaire for smoking urges	Change in psychiatric symptoms (AIMS, BDI, PANSS, WEPS)
George 2008 [21] RCT	58 clinically stable outpatients with DSM IV schizophrenia or schizoaffective disorder on a stable dose of antipsychotic medication and smoking ≥10 cigarettes per day with expired CO > 10 ppm and scored ≥7 on the contemplation ladder United States 60% male, 48% white.	1. Bupropion + manualised group behavioural therapy + NRT patch (21 mg) 2. Placebo + manualised group behavioural therapy NRT patch (21 mg) 150 mg per day days 1–3 and 150 mg 2 x day thereafter	7 day point prevalence abstinence at week 10, and 26 verified by expired CO <10 ppm. 4 week continuous abstinence at week 10.	Change in psychiatric symptoms (BDI, PANS) Adverse events
Weinberger 2008 [22] RCT	5 clinically stable DSM-IV Bipolar disorder I outpatients smoking ≥10 cigarettes per day with expired CO ≥ 10 ppm United States 40% male, 100% white.	1. Bupropion + manualised group behavioural therapy 2. Placebo + manualised group behavioural therapy (Days 1–3 75 mg 1 x day, days 4–7 150 mg 1 x day and 150 mg 2× day thereafter)	Abstinence at 10 weeks verified by expired CO <10 ppm.	Change in psychiatric symptoms (YMRS, BDI, Ham-D) Adverse events
Weiner 2012 [25] RCT	41 clinically stable adult outpatients with DSM IV schizophrenia or schizoaffective disorder who Smoke ≥10 and scored ≥ x on FTND United States 79% male. 72% white.	1. Bupropion + group support programme 2. Placebo + group support programme (Days 1–3150 mg 1 x day and 150 mg 2× day thereafter)	Complete abstinence at 15 weeks defined by expired CO < 10 ppm at last 4 study visits. Complete abstinence at 6 months and 12 months self-report verified by CO < 10 ppm 7 day point prevalence abstinence at 15 weeks verified by CO < 10 ppm FTND	Change in psychiatric symptoms (BPRS, SANS, SAS) Adverse events
Tidey 2011 [24] RCT	57 clinically stable adult outpatients with DSM IV schizophrenia or schizoaffective disorder on a stable dose of psychoactive medication who Smoke ≥20 cigarettes per day and scored ≥6 on FTND and ≥4 on the contemplation ladder indicating some interest in quitting smoking United states 71% male, 75% white.	1. Contingent + Bupropion (150 mg per day days 1–3 and 150 mg 2 x day thereafter) 2. Contingent + placebo 3. Bupropion (150 mg per day days 1–3 and 150 mg 2 x day thereafter) + non-contingent 4. Placebo +non contingent Non contingent = $25 dollar store card Contingent = $25 store card plus bonuses	Cotinine in urine CO breath measure Number of cigarettes per day At weeks 1,2,3 and 4	Change in psychiatric symptoms (PANSS, UPDRS, AIMS)
Varenicline studies				
Chengappa 2014 [31] RCT	60 adult outpatients with DSM-IV bipolar disorder on a stable dose of medication. Smoking ≥10 cigarettes per day with expired CO ≥ 10 ppm United States Ethnicity and gender not reported.	1. Varenicline + smoking cessation counselling 2. Placebo + smoking cessation counselling1 × 0.5 mg per day days 1–3, 0.5 mg 2× per day days 4–7 then 1 mg 2× per day thereafter	7 day point prevalence smoking abstinence verified by expired CO <10 ppm at 12 weeks and 24 weeks Continuous 4 week abstinence at 12 weeks	Change in Psychiatric symptoms (YMRS, MADRS, HARS, CGI) Adverse events
Smith 2016 [35] RCT	87 adult inpatients or outpatients with DSM IV schizophrenia or schizoaffective disorder who smoke at least 6 cigarettes per day or in the case of inpatients had flouted the smoking ban on several occasions. United States, Israel and China 85% male, 31% white.	1. Varenicline + smoking prevention counselling 2. Placebo + smoking prevention counselling 1 × 0.5 mg per day days 1–3, 0.5 mg 2× per day days 4–7 then 1 mg 2× per day thereafter	Self-reported number of cigarettes smoked per day Expired CO, cotinine levels and urges to smoke.	Change in psychiatric symptoms (PANSS, SANS, Calgary Depression Scale) Adverse events
Weiner 2011 [25] RCT	9 Clinically stable adult outpatients with DSM IV schizophrenia or schizoaffective disorder for 3 years who smoke ≥10 and scored ≥4 on FTND. United States Ethnicity and gender not reported.	1. Varenicline (1 mg 2× day) + individual smoking cessation counselling (ALA) 2. Placebo + individual smoking cessation counselling (ALA)	Smoking cessation at 12 weeks defined by expired CO < 10 at last 4 study visits. Change in CO	Change in psychiatric symptoms (BPRS) Adverse events
Williams 2012 [27] RCT	128 adult outpatients with DSM IV schizophrenia or schizoaffective disorder with stable symptoms who Smoke ≥15 and scored ≥7 on the contemplation ladder indicating a willing ness to quit in the next month and with no smoking abstinence in the last 3 months United States and Canada 76% male, 59% white.	1. Varenicline 2. Placebo 1 × 0.5 mg per day days 1–3, 0.5 mg 2× per day days 4–7 then 1 mg 2× per day thereafter	7 day point prevalence abstinence at 12 and 24 weeks verified by expired CO <10 ppm. Number of cigarettes per day	Change in psychiatric symptoms (SAS, C-SSRS, CGI, PANSS) Adverse events
Wu 2012 [37] RCT	5 psychiatrically stable DSM-IV bipolar disorder I or II on a stable dose of mood stabliser, smoking ≥10 cigarettes per day. Outpatients 40% male, 100% white	1. Varenicline (1 mg 2× day) + smoking cessation counselling (group) 2. Placebo + smoking cessation counselling (group)	Smoking cessation verified by expired CO >10 ppm at 10 weeks and 6 months	Adverse events
Nicotine Replacement Therapy (NRT) studies				
Chen 2013 [29] RCT	184 adult inpatients who were regular daily smokers with DSM-IV schizophrenia or schizoaffective disorder with stable symptoms. Taiwan93% male, ethnicity not stated.	1. High dose NRT (31.2 mg for 4 weeks then 20.8 mg for 4 weeks) 2. Low dose NRT (20.8 mg for 8 weeks)	7 day point prevalence self report verified by expired CO <10 ppm at 5 weeks and 8 weeks Number of cigarettes smoked per day FTND	Change in psychiatric symptoms (PANSSS, SAS)
Dalak 1999 [11] RCT (within subject crossover)	19 male veteran outpatients with DSM III schizophrenia, schizoaffective disorder Smoking ≥20 cigarettes per day on a stable antipsychotic regime. United States 100% male, 60% white.	1. Nicotine patches (22 mg per day) 2. Placebo patches	Nicotine blood level Expired CO Cotinine blood level	Change in psychiatric symptoms (BPRS, SANS, HAM-D) Adverse events
Gallagher 2007 [20] RCT	181 stable adult outpatients with DSM-IV schizophrenia or schizoaffective disorder, smoking ≥10 cigarettes per day for 3 years or more with expired CO ≥ 10 ppm after 15 min smoke free. United States 52% male, 76% white.	1. Contingent reinforcement (up to $480) 2. Contingent reinforcement (up to $480) + NRT patch (21 mg) 3. Self-quit group	Smoking cessation at week 20 and week 36 (Cotinine ≤15 ng/ml or expired CO ≤ 10 ppm) FTND	Change in psychiatric symptoms (BSI)

*AIMS* abnormal involuntary movement scale; *ALA* American Lung Association; *BDI* Beck Depression Index; *BPRS* Brief Psychiatric Rating Scale; *CBT* cognitive behaviour therapy; *CGI*-*S* Clinical Global Impression- Severity of Illness Scale; *CO* carbon monoxide; *C*-*SSRS* Columbia Suicide Severity of Illness Scale; *DSM* Diagnosis and Statistical Manual; *Ham*-*D* Hamilton Depression Rating Scale; *FTND* Fagerstrom Test fro Nicotine Dependence; *ICD* International Classification of Disease; *MADRS* Montgomery-Asberg Depression Scale; *MNWS* Minnesota Withdrawal Scale-Revised; *NRT* nicotine replacement therapy; *PANSS*: Positive and Negative Syndrome Scale; *SANS* Scale for Assessment of Negative Symptoms; *SAS* Simpson Angus Scale; *SF*-*12* 21 item Short Form Survey on general functioning; *SRP* Sustained Release Preparation; *p.p.m.* parts per million; *STAI* State Trait Anxiety Inventory; *UPDRS* Unified Parkinson’s Disease Rating Scale; *WEPS* Webster Extrapyramidial Movement Scale; *WISDM* Wisconsin Inventory of Smoking Dependence Motives; *YMRS* Young Mania Rating Scale

Sixteen of the studies were conducted in the United States, two in Australia, one in Taiwan, one in England one in the United States, Israel and China and one in the United States and Canada. In four studies the country was not clearly stated.

The majority of the studies recruited participants with schizophrenia or schizoaffective disorder (*n* = 21), with three studies recruiting participants with bipolar disorder, and two studies included participants with schizophrenia, schizoaffective disorder or bipolar disorder. In eight of the studies it was a study requirement that the participants had stable symptoms, in three studies it was a requirement that participants were on a stable dose of medication and in six studies it was a requirement that participants has stable symptoms and were on a stable dose of medication. Nine studies did not state whether the participants were clinically stable or were on a stable dose of medication.

In just over half of the studies the participants had expressed a willingness to quit smoking (*n* = 12), in one study participants were excluded if they were planning on quitting in the next 30 days [36] and in the remaining 12 studies participants’ views on quitting were not stated. No study stated that it was recruiting participants with no interest in quitting smoking.

Nine of the studies used an intention to treat analysis, one used a per protocol analysis [36] and 16 studies did not report whether or not they used an intention to treat analysis.

### Description of the interventions

The included studies covered a range of interventions (Table 1). Nine studies explored the effects of the prescription of bupropion, six studies the prescription of varenicline and one study the prescription of nicotine replacement therapy (NRT). The varenicline studies all followed a standard dosing schedule whereas the dose in the bupropion studies ranged from 150 mg once per day to150 mg twice per day. Five studies explored the effects of a specialist smoking cessation programme for people with SMI and three studies investigated the effects of contingent reinforcement (i.e., providing people with cash incentives if they had remained abstinent from smoking at defined time points).

Of the nine trials (involving 306 participants in total) which explored the effects of bupropion, five tested bupropion plus group therapy versus placebo plus group therapy [13, 14, 17, 22, 26], two tested bupropion plus group therapy plus NRT versus placebo plus group therapy plus NRT [19, 21] one tested bupropion plus smoking cessation counselling versus placebo plus smoking cessation counselling [30]. The final study employed a factorial design testing contingent plus bupropion versus non-contingent plus bupropion versus contingent plus placebo versus non-contingent plus placebo [24]. Tidey did not report abstinence therefore was not included in the meta-analysis.

The addition of varenicline to a range of interventions in the control arm was tested in six trials (313 participants in total). Of these six trials, four tested varenicline plus smoking cessation counselling versus placebo plus counselling [30, 31, 35, 37], one tested varenicline plus group therapy versus placebo plus group therapy [25], and one tested varenicline versus placebo [27].

Five studies explored the effects of a smoking cessation programme designed for people with SMI (638 participants): two studies compared the smoking cessation programme to usual care [18, 33], one explored a specialist programme plus NRT versus a standard smoking programme plus NRT [12], one study compared a specialist programme with medication management [23], and one study compared motivational interviewing with personalised feedback with interactive education with no personalisation [36].

Smoking cessation counselling, whether part of the intervention being tested or part of the control arm, consisted of a range of behaviour change techniques delivered in a variety of formats e.g. face-to-face one-to-one sessions, face-to-face group sessions or one-to-one sessions delivered via telephone. It is important to note that in the trials of varenicline and bupropion, where smoking cessation counselling was delivered, the same programme was delivered in both the medication (varenicline or bupropion) arm of the trial as in the usual care arm of the trial. Therefore it is unlikely that the smoking cessation counselling component of the study had any bearing on the study results. In the majority of the trials the exact content, in terms of the behaviour change techniques employed in the smoking cessation counselling, was insufficiently described.

No studies were identified exploring the effectiveness of very brief advice or the effectiveness of electronic cigarettes.

### Methodological quality

Table 2 Summarises the risk of bias in the included studies. Overall the studies were at high risk or unclear risk of bias aside from Smith 2015 [34] and Smith 2016 [35] which were both at low risk of bias. Overall there was a lack of detail given in the descriptions of key study design features which has led to studies being deemed at an unclear risk of bias. For those studies that were assessed as having an unclear risk of bias the issue may be with the reporting as opposed to actual study conduct. The risk of bias was assessed by two reviewers and there were only few disagreements which were simply resolved by discussion until consensus was reached. Discussion with 3rd reviewer not necessary in any of the instances.

**Table 2:** Risk of bias of included studies

	Adequate sequence generation	Allocation concealment	Blinding of participants and personnel	Blinding of outcome assessment	Incomplete outcome data addressed	Free of selective reporting	Free of other bias	Overall
Baker 2006 [18]	Unclear	High Risk	High Risk	Low Risk	Low Risk	Low Risk	Low risk	High risk
Baker 2015 [32]	Unclear	Unclear	High Risk	Low Risk	High Risk	Low Risk	Low risk	High
Chen 2013 [29]	Unclear	Unclear	Unclear	Unclear	Low Risk	Unclear	Low risk	Unclear
Chengappa 2014 [31]	Unclear	Unclear	Low Risk	Low Risk	Unclear	Low risk	Low risk	Unclear
Dalak 1999 [11]	Unclear	Unclear	Unclear	Unclear	Unclear	High risk	High risk	Unclear
Evins 2001 [13]	Unclear	Unclear	Unclear	Unclear	Low Risk	Unclear	Low risk	Unclear
Evins 2005 [17]	Unclear	Unclear	Unclear	Unclear	Unclear	Unclear	High risk	Unclear
Evins 2007 [19]	Unclear	Unclear	Unclear	Unclear	Unclear	Unclear	Low risk	Unclear
Fatemi 2013 [30]	Unclear	Unclear	Unclear	Unclear	Unclear	Unclear	Unclear	Unclear
Gallagher 2007 [20]	Unclear	Unclear	High Risk	High Risk	High risk	Unclear	Low risk	High
George 2000 [12]	Unclear	Unclear	High Risk	Unclear	Unclear	Unclear	High risk	High
George 2002 [14]	Unclear	Unclear	Low Risk	Low Risk	Low risk	Unclear	Low risk	Unclear
George 2008 [21]	Unclear	Unclear	Unclear	Unclear	High risk	High risk	Low risk	High
Gilbody 2015 [33]	Low Risk	Low Risk	High Risk	High Risk	Low Risk	High Risk	Low risk	High
Steinberg 2003 [15]	Unclear	Unclear	High Risk	Low Risk	High Risk	Unclear	Low risk	High
Tidey 2011 [24]	High Risk	High Risk	Low Risk	Low Risk	High Risk	Unclear	High risk	High
Weinberger 2008 [22]	Unclear	Unclear	Unclear	Unclear	High Risk	Unclear	High risk	High
Weiner 2011 [25]	Unclear	Unclear	Unclear	Unclear	Unclear	Unclear	Unclear	Unclear
Weiner 2012 [26]	Unclear	Unclear	Unclear	Unclear	Low risk	Unclear	Low risk	Unclear
Williams 2010 [23]	Unclear	Unclear	High Risk	Low Risk	High Risk	Unclear	High risk	High
Williams 2012 [27]	Unclear	Unclear	Low Risk	Low Risk	Low Risk	Low Risk	Unclear	Unclear
Wing 2012 [28]	Unclear	Unclear	Unclear	Unclear	Unclear	Unclear	Unclear	Unclear
Wu 2012 [37]		Unclear	Unclear	Unclear	Unclear	Unclear	Unclear	Unclear
Steinberg 2016 [36]	Unclear	Unclear	High risk	Low risk	Low risk	Unclear	High risk	High
Smith 2015 [34]	Low risk	Low risk	Low risk	Low risk	Unclear	Unclear	Low risk	Low risk
Smith 2016 [35]	Low risk	Low risk	Low risk	Low risk	Low risk	Low risk	Unclear	Low risk

### Smoking abstinence

**Fig. 2:**
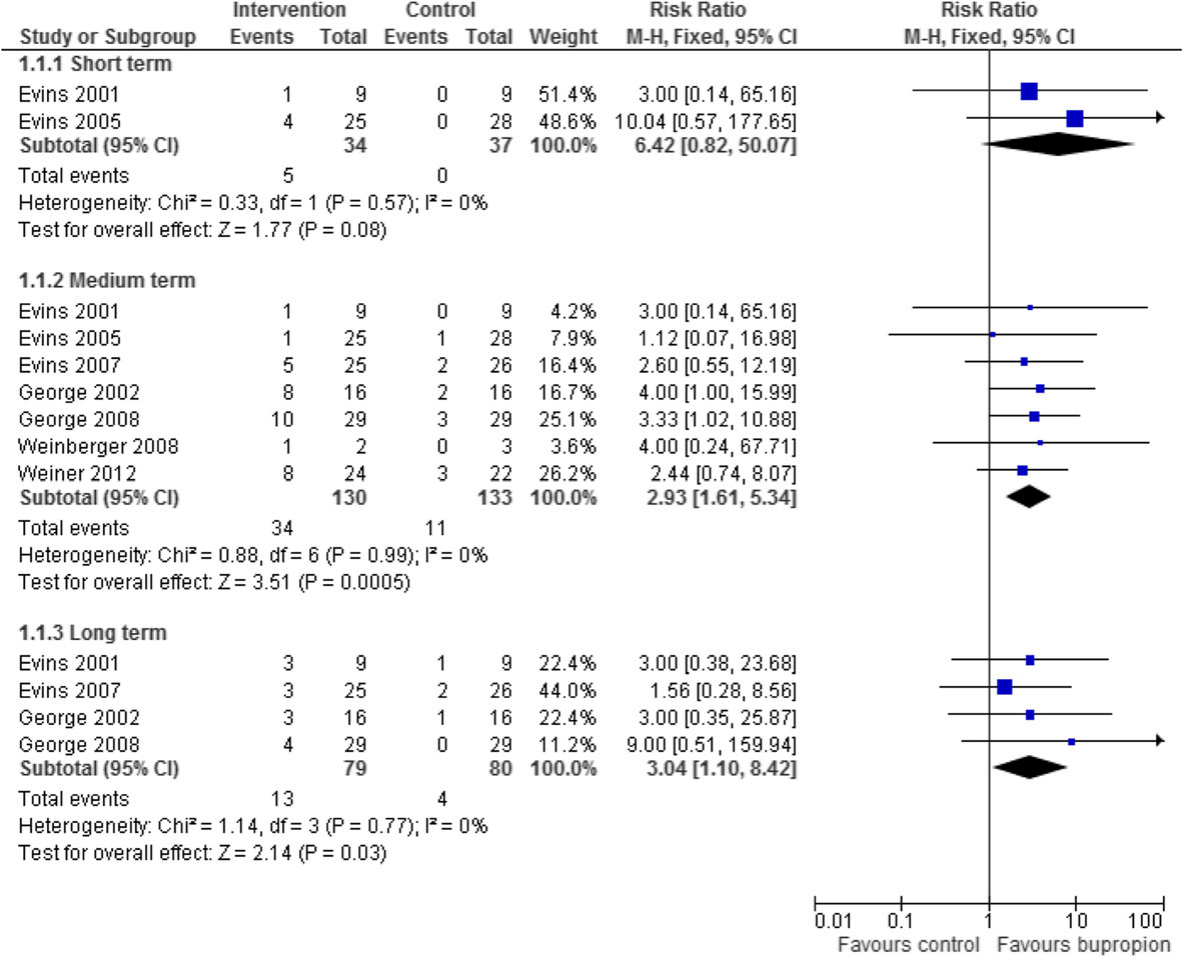
Addition of bupropion

**Fig. 3:**
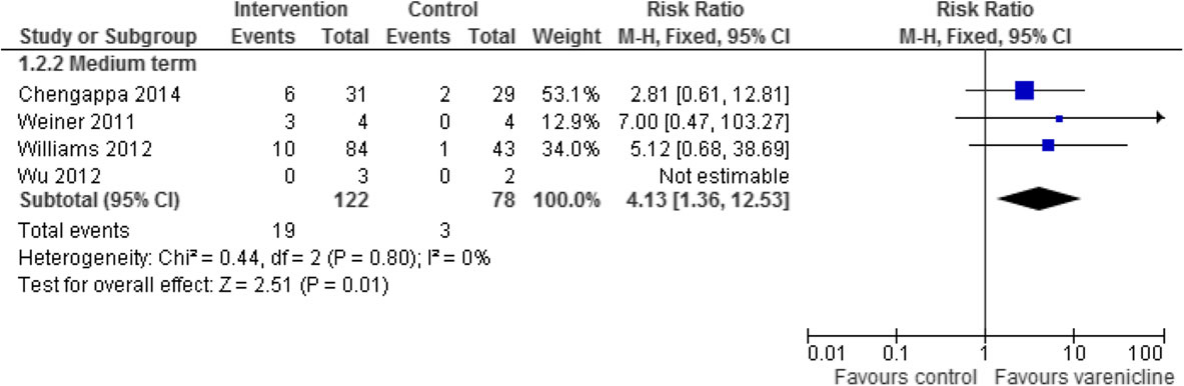
Addition of varenicline

**Fig. 4:**
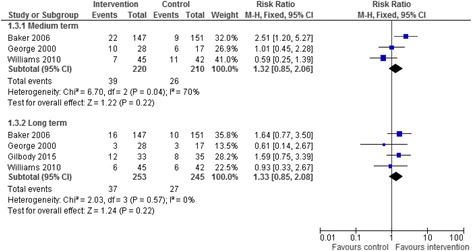
Addition of specialist smoking cessation programme

Risk ratio (pooled) for point prevalence abstinence at short, medium and long term for studies exploring the addition of bupropion (Fig. 2), varenicline (Fig. 3) and a specialist smoking intervention for people with SMI (Fig. 4) were calculated. Funnel plots are not included in this review because we identified less than 10 studies eligible for inclusion in the meta-analyses.

### Bupropion versus placebo

Eight trials that tested the addition of bupropion to a range of interventions in the control arm reported abstinence data. These studies were pooled to judge whether the addition of bupropion offered any additional benefit (Fig. 2). Pooling this data using a fixed-effects meta-analysis showed that the addition of bupropion improved quit rates significantly in the medium term and long term but not in the short term (short term RR = 6.42 95% CI 0.82–50.07; medium term RR = 2.93 95% CI 1.61–5.34; long term RR = 3.04 95% CI 1.10–8.42). The median duration of the short term comparison was four weeks, 3.5 months for the medium term comparison, and 11.75 months for the long term comparison. There was no evidence of between study heterogeneity (I^2^ = 0%).

### Varenicline versus placebo

Five of these studies were pooled to evaluate whether the addition of varenicline offered any additional benefit (Fig. 3). Pooling this data using a fixed-effects meta-analysis showed that the addition of varenicline improved quit rates significantly in the medium term (RR = 4.13 95% CI 1.36–12.53), median time-point six months. None of these five studies gave long term quit data. There was no evidence of between study heterogeneity (I^2^ = 0%). Participants in these studies received varenicline for between eight and 12 weeks. Removing the monotherapy study [27] from the meta-analysis did not substantially change the results and there was no overall change in heterogeneity (RR = 3.62 95% CI 0.68–38.69).

### Specialist smoking cessation programme

The results from the studies exploring smoking cessation interventions were mixed in terms of results when compared to those exploring the effectiveness of smoking cessation medication. Whilst some studies reported positive findings others reported negative findings. This may be due to differences in the smoking cessation intervention being tested. It may be that some interventions or components of interventions are more effective than other smoking cessation interventions, however this cannot be certain. The setting, delivery mode and who delivers the intervention may also have some influence of the effectiveness of the intervention.

Four studies gave abstinence data, three of which gave medium term data and long term data and one gave long terms data only. These studies were pooled to assess whether a specialist programme offered any additional benefit (Fig.4). Pooling this data using a fixed-effects meta-analysis showed that there was no evidence of benefit for the specialist smoking cessation programme in the medium term (RR = 1.32 95% CI 0.85–2.06) or in the long term (RR = 1.33 95% CI 0.85–2.08). Median duration of comparison was six months in the medium term and 12 months in the long term. None of these five studies gave short term quit data. There was no evidence of between study heterogeneity (I^2^ = 0%).

### Secondary outcomes

#### Change in psychiatric symptoms

Of the included studies, 22 used one or more validated symptom scales to ascertain whether psychiatric symptoms had altered during the course of the trial (Table 3). None of the studies that tested outcomes for significance found any significant worsening of psychiatric symptoms in the intervention group and only one study found a significant worsening of cognitive score in the intervention group compared to placebo [17]. Therefore it does not appear that smoking cessation interventions worsened psychiatric symptoms however due to heterogeneity between the symptom scales and time points used no meta-analysis was conducted.

**Table 3:** Outcomes

	Change in BMI	Change in psychiatric symptoms	Adverse events	Quit rate (%) intervention (I) control (C)
Complex interventions				
Baker 2006 [18, 38] (including data from Baker 2010)	Not reported	Time-points: 4 months, 7 months, 13 months CDI: significantly lower score for intervention group *p* < 0.001 at all time-points BPRS: not significant at any time point SF-12 (mental): significantly lower score for intervention group *p* < 0.001 at all time-points STAI: significantly lower for intervention group *p* < 0.001 at 7 months	Not reported	4 months I: 22/147 (15.0) C: 9/151 (6.0) 7 months I: 14/147 (9.5) C: 6/151 (4.0) 13 months I: 16/147 (10.9) C: 10/151 (6.6) 4 years I: 13/147 (8.8) C: 17/151 (11.3)
Baker 2015 [32]	Not reported	Time point 3.75, 12 months BPRS, BDI, GAF, SF-12 not significant	Not reported	3 months I: 13/122(10.7) C: 13/113 (11.5) 12 months I: 8/122 (6.6) C: 7/113 (6.2)
George 2000 [12]	Not reported	Time-points: 3 months, 8.5 months AIMS, BDI, PANSS, WEPS: not significant	Not reported	3 months I: 10/28 (35.7) C: 6/17 (35.3) 8.5 months I: 3/28 (10.7) C: 3/17 (17.6)
Gilbody 2015 [33]	Change in BMI not reported. Mean BMI at baseline and 12 month reported.	Time points 1,6,12 months PHQ-9, EQ-5D, SF-12 mental reported but not tested for significance	21 events of which 12 SAEs, 10 in intervention 2 in usual care	12 months I: 12/33 (36.3) C: 8/35 (22.9)
Smith 2015 [34]	Not reported	Time point after final session PANSS and PYCHRATS no significant differences	15 AEs in active treatment arm and 16 in sham treatment arm	Abstinence not reported
Steinberg 2003 [15]	Not reported	Not reported	Not reported	Abstinence not reported
Steinberg 2016 [36]	Not reported	Not reported	Not reported	1 month I: 8/49 (16.3) C: 5/49 (10.2)
Williams 2010 [23]	Not reported	Time-point 3 months BDI and PANSS positive and negative not significant	Not reported	3 months I: 7/45 (15.6) C: 11/42 (26.2) 6 months I: 7/45 (15.6) C: 8/43 (18.6) 12 months I: 6/45 (13.3) C: 6/43 (14.0)
Wing 2012 [28]	Not reported	No detail on secondary outcomes given	Not reported	Abstinence not reported
Bupropion studies				
Evins 2001 [13, 16] (including data from Evins 2004)		Time-points 3 months, 6 months AIMS, SANS, SAS: not significant BPRS (total): significant decrease intervention group 0–3 months (*p* = 0.03) and 3–6 months (*p* = 0.02) BPRS (+ve symptoms): significant decrease intervention group 0–3 months (*p* = 0.03). Not significant 3-6 m. HAM-D: significant increase for placebo group 0–3 months (*p* < 0.01). Not significant 3-6 m. HAS: not significant	No adverse events	1 months I: 3/9 (33.3) C: 1/9 (11.1) 3 months I: 1/9 (11.1) C: (0/9) (0.0) 6 months I: 1/9 (11.1) C: 0/9 (0.0) 24 months I: 2/9 (22.2) C: 2/9 (22.2)
Evins 2005 [17]	Not reported	Time-points 3 months Barnes Akathisia Scale: not significant HAM-A, HAM-D, SANS, SAS, WEPS, PANSS (total): not significant PANSS (subscale); significant increase in excitement score placebo versus intervention group (*P* = 0.017) Significant decrease cognitive score intervention versus placebo (*P* = 0.029) Other subscales not significant	3 events requiring withdrawal, 1 in the intervention, 2 group unknown	1 months I: 9/25 (36.0) C: 2/28 (7.0) 3 months 4/25 (16.0) c: 2/28 (0.0) 3.5 months I: 2/25 (8.0) C: 1/28 (3.6) 6 months I: 1/25 (4.0) C: 1/28 (3.6)
Evins 2007 [19]	Not reported	Time-points: 3 months AIMS, BDI, SANS, STAI, HAM-D, PANSS: not significant Barnes Akathisia Scale: significantly lower in intervention group (*P* = 0.005) SAS: significantly lower score in the intervention group (*P* = 0.016)	No SAEs	2 months* I: 13/25 (52.0) C:5/26 (19.2) 3 months* I: 9/25 (36.0) C: 5/26 (19.2) 6 months* I: 5/25 (20.0) C: 2/26 (7.7) 15 months* I: 3/25 (12.0) C: 2/26 (7.7)
Fatemi 2013 [30]	Not reported	Time point: 3 months Significant positive correlation between serum cotinine levels and BPRS total score (*p* = 0.014), BPRS +ve subscale score (*p* = 0.002), SAPS total composite score (*p* = 0.02) and SAPS delusion subscale score (*p* = 0.013)	Not fully reported	Abstinence not reported
George 2002 [14]	Not reported	Time-points 2.5 months, 8.5 months AIMS, BDI, WEPS: not significant PANSS: significant decrease in intervention group for negative symptoms (*P* < 0.05; general positive subscales not significant	Not reported	2.5 months I: 8/16 (50.0) C: 2/16 (12.5) 8.5 months I: 3/16 (18.8) C: 1/16 (6.3)
George 2008 [21]	Not reported	Time-points: 2.5 months, 6.75 months BDI, PANSS: not significant	No SAEs	2.5 months I: 10/29 (34.5) C: 3/29 (10.3) 6.75 months I: 4/29 (13.8) C: 0/29 (0.0)
Weinberger 2008 [22]	Not reported	No details given on secondary outcomes	Not fully reported	2.5 months I: 1 /2 (50.0) C: 0/3 (0.0)
Weiner 2012 [26]	Not reported	Time-points: 2 weeks, 1 month, 2 months and 3.5 months BPRS, SANS: not significant	5 SAEs in the intervention group and 2 in the placebo group	3.5 months I: 8/24 (33.3) C: 3/22 (13.6)
Tidey 2011 [24]	Not reported	Time-points 1,2, 3, 4 weeks PANSS, UPDRC ad AIMS not significant	Not fully reported incidence of specific AEs reported but not all	Abstinence not reported
Varenicline studies				
Chengappa 2014 [31]	Mean weight gain	Time-points 3, 6 months Scores for MADRS, YMRS, HARS and CGI reported but not tested for significance.	6 SAEs in the intervention group and 4 in the placebo group	3 months I: 15/31 (48.4) C: 3/29 (10.3) 6 months I: 6/31(19.4) C: 2/29 (6.9)
Smith 2016 [35]	Not reported	Time-point 8 weeks Scores for PANSS, and SANS not significant when corrected for multiple comparisons.	Comparisons made between number of AEs in both groups. Concluded that no AE involving emergent psychiatric symptoms could be attributed to varenicline.	8 weeks I:7/42 (16.7) C: 4/45 (8.9)
Weiner 2011 [25]	Not reported	Time-points 3 months BPRS +ve items, anxiety/depression not significant	8 side effects in the intervention group 2 in the placebo group	4 months I: 3 /4 (0.75) C: 0/4 (0.0)
Williams 2012 [27]	Not reported	Time-points: 3, 6 months PANSS not significant	9 SAEs in the intervention group and 4 in the placebo group	3 months I: 16/84 (19.0) C: 2/43 (4.7) 6 months I: 10/84 (11.9) C: 1/43 (2.3)
Wu 2012 [37]	Not reported	Time-points 2.5 months Psychiatric symptoms not significantly changed	Not fully reported	2.5 months I: 1/3 (33.3) C: 0/2 (0.0) 6 months I: 0/3 (0.0) C: 0/2 (0.0)
NRT studies				
Chen 2013 [29]	Not reported	Time-points: 2 months PANSS, SAS not significant	Not reported	2 months I: 1/92 (1.1) C: 4/92 (4.3) I = high dose C = low dose
Dalak 1999 [11]	Not reported	Time-points: day 2 AIMS: significantly increased score intervention group day 2 (*p* < 0.05) BPRS, HAM-D, SANS, SAS: not significant	Assessment for signs of nicotine toxicity none reported	Abstinence not reported
Gallagher 2007 [20]	Not reported	Time points 5, 9 months BSI not significant	Not reported	5 months Ia**: 23/60 (38.3) Ib***: 25/60 (41.7) C: 3/60 (5.0) 8.5 months Ia: 22/60 (36.7) Ib: 26/60 (43.3) C: 5/60 (8.3)

**Ia = contingent reinforcement *** Ib = Contingent reinforcement plus NRT

Only one study that included participants with bipolar disorder reported on the significance of any change in psychiatric symptoms (not significant). The rest of the studies that reported secondary outcome included participants with schizophrenia and schizoaffective disorder.

#### Change in BMI

Change in BMI was not routinely measured in the included studies and only two studies listed BMI as one of their outcomes [31, 33]. Of these only one study reported change in BMI therefore no meta-analysis was conducted.

#### Adverse events

Of the included studies 14 included some reporting of adverse events (Table 3), although in four of these studies this was not fully reported. No standardised method for reporting adverse events was used and some studies differentiated between serious adverse events and adverse events whereas some did not.

#### Cost effectiveness

Only one study [33] set out to explore the cost-effectiveness of the intervention. This study demonstrated that it was feasible to carry out a cost-effectiveness analysis of a bespoke smoking cessation intervention compared to usual care however as it was a pilot study it was not sufficiently powered for any firm conclusions could be drawn.

## Discussion

Since our previous review there has been an increase in the evidence base of smoking cessation interventions for people with SMI. Previously we identified seven studies meeting the inclusion criteria, in this review we have included 26 studies, 19 more than our previous review, indicating that this is a rapidly developing field. Despite the increase in the number of studies exploring the effectiveness of smoking cessation interventions for people with SMI, the studies are still generally of a small size and underpowered to detect a difference between the intervention and control. Overall studies were at high or unclear risk of bias with only two of the most recent studies being at low risk of bias [34, 35].

In line with the results of our previous review, this updated review indicates that people with SMI can quit smoking and the same interventions that work for people in the general population work for people with SMI e.g. the use of varenicline, bupropion or NRT to support a quit attempt. The addition of bupropion gives a similar risk ratio at both medium and long term to that of our previous review [7]. In our previous review we calculated an RR = 2.76 (95% CI 1.48–5.16) CI 1.10–8.42) compared to 3.04 (95% CI 1.10–8.42) for long term point prevalence. For varenicline our review showed a slight increase in RR compared to a recent Cochrane review [39] where the RR = 2.27 (95% CI 2.02–2.55) whilst our meta-analysis gave a medium term RR of 2.93 (95% CI 1.61–5.34). A recent review of the effectiveness of varenicline in people with SMI which had slightly different inclusion criteria to our review also concluded that varenicline was clinically superior to placebo in helping people with SMI [40]. Due to the unclear or high risk of bias of 24 of the 26 included studies in our review our results need to be interpreted with some caution.

Point prevalence absolute quit rates at the final time-point for intervention groups ranged from 1.1 to 75.0%, and for control groups ranged from 0.0 to 22.9%. In addition quitting smoking did not appear to worsen participants’ mental state. In terms of varenicline and bupropion our review indicates that both medications appear to be effective in the medium terms as an aid to smoking cessation. A recent large trial comparing outcomes of people with psychiatric disorder has also found varenicline and bupropion to be effective with no increase in neuropsychiatric events [41], however this study was not eligible for inclusion in our review as the psychiatric cohort was not limited to people with SMI. The effectiveness of behavioural interventions in helping people with SMI to quit smoking is currently unclear and is the subject of on-going study [42].

We identified two studies [29, 35] that included patients in an inpatient setting, however the majority of the studies were conducted in a psychiatrically stable population and it is therefore unclear as in our previous review how far these findings are generalisable to an acutely unwell population. It is important that further studies are conducted into what works in an acutely unwell population.

The use of e-cigarettes has been increasing in recent years [43] and a Cochrane review was conducted in 2016 exploring their effectiveness as a smoking cessation aid [44]. E-cigarettes have been shown to have a similar effect on quit rate as NRT [45]. However we did not identify any RCTs that explored the use of e-cigarettes as a smoking cessation aid for people with SMI. A subgroup analysis of people who took part in the ASCEND trial was conducted analysing the results for people with mental disorders however this was not limited to SMI [46]. This subgroup analysis indicated that e-cigarettes appear to be as effective in people with mental disorders as those without mental disorders. This topic deserves further research and there is a need for future trials of electronic cigarettes as an aid to smoking cessation amongst people who use mental health services.

Only one study investigated the cost effectiveness of a smoking cessation intervention and this was a pilot study so no clear conclusions could be drawn [33]. More trials are needed with a prospective cost effectiveness analysis. In addition how an intervention may fit into existing service structures needs to be explored.

Only one study reported change in body weight and this was reported as mean change in BMI [31]. Given that weight gain is associated with the prescription of antipsychotic medication [47] and the health implications of obesity it is important that weight change is recorded and reporting in clinical trials. A recent systematic review demonstrated that whilst the mean increase in body mass 12 months after stopping smoking is four to five kilograms there was a wide variation in body mass change [48] (16% of participants had a reduced mass and 13% gained more than 10 kg).

The reporting of adverse events was not standardised. In 12 of the studies included in this review no details of adverse or serious adverse events were reported. It is important that adverse events are clearly reported as per the CONSORT guidelines [49] to allow a judgment to be made as to whether or not a pharmaceutical smoking cessation aid is suitable for people with SMI.

### Strengths and limitations

A limitation of this review is that it only included articles that were written in English and this could have resulted in the exclusion of potentially important studies. The fact that all the titles and abstracts were not double screened is a possible limitation however the fact that both authors who screened the initial 10% of titles and abstracts were in agreement over which studies should go forward to full text review reduces the possibility that potentially suitable studies were missed. In addition reference lists of previous reviews of smoking cessation strategies were searched. There is currently a paucity of e-cigarette research. This is a technology that is rapidly evolving and where there has been uptake in the use of e-cigarettes in advance of randomised trials being conducted. However, a strength of this review compared to our previous review is that it includes the use of e-cigarettes as a smoking cessation aid.

Due to the heterogeneity of the scales used to assess psychiatric symptoms it was not possible to conduct a detailed analysis of the results or a meta-analysis. We have therefore summarised whether or not studies found a significant change in psychiatric symptoms and concluded that no significant worsening was found on giving up smoking.

It is possible that the results of this review are at risk of publication bias. To minimise the possibility of publication bias we checked trial registries to determine whether there were any trials registered that had not been published. Funnel plots are not included in this review because we identified less than 10 studies eligible for inclusion in the meta-anayses.

### Recommendations for future research

It is currently unclear what proportion of people with SMI will engage with a smoking cessation intervention and trials are needed that will explore the use of very brief advice to encourage people with SMI to seek help with smoking. It is also recommended that the use of e-cigarettes as a smoking cessation aid for people with SMI be explored in future high quality RCTs.

## Conclusions

Despite evidence for the effectiveness of smoking cessation interventions for people with SMI the percentage of people with SMI who smoke in the UK still remains higher than the percentage of people without SMI who smoke.

In addition to our previous findings regarding the effectiveness of bupropion in helping people with SMI to quit smoking there is now trial based evidence to demonstrate that varenicline appears to be effective in helping people with SMI to quit smoking.

## Additional file

Additional file 1: Search strategy, Description: example search strategy. (DOCX 13 kb)

## Abbreviations

BMI: body mass index
CI: confidence interval
CO: carbon monoxide
NRT: nicotine replacement therapy
RCT: randomised controlled trial
RR: risk ratio
SMI: severe mental ill health
