# Supplementary material for: Smoking cessation in severe mental ill health: what works? an updated systematic review and meta-analysis
Source: BMC Psychiatry. 2017 Jul 14;17:252. doi: 10.1186/s12888-017-1419-7 (PMC5513129; doi:10.1186/s12888-017-1419-7)
Supplement: Additional file 1: — Search strategy, Description: example search strategy. (DOCX 13 kb) [file 12888_2017_1419_MOESM1_ESM.docx]

Additional file one: Search strategy

Host: OvidSP 
Dates searched: Unclear- Ongoing 
Search strategy: 
1. exp Clinical Trial/

2. exp Randomized Controlled Trial/

3. exp Double-Blind Method/

4. exp Single-Blind Method/

5. exp Cross-Over Studies/

6. randomized controlled trial.pt.

7. clinical trial.pt.

8. controlled clinical trial.pt.

9. (clinic$ adj2 trial).mp.

10. (random$ adj5 control$ adj5 trial$).mp.

11. (crossover or cross-over).mp.

12. ((singl$ or double$ or trebl$ or tripl$) adj (blind$ or mask$)).mp.

13. randomi$.mp.

14. (random$ adj5 (assign$ or allocat$ or assort$ or receiv$)).mp.

15. or/1-14

16. exp Schizophrenia/

17. exp Paranoid Disorders/

18. schizo$.mp.

19. hebephreni$.mp.

20. psychotic$.mp.

21. psychosis.mp.

22. psychoses.mp.

23. ((chronic$ or sever$) adj2 mental$ adj2 (ill$ or disorder$)).mp.

24. exp Bipolar Disorder/

25. or/16-24

26. 15 and 25

27. smoking cessation.mp. or exp Smoking Cessation/

28. "Tobacco‐Use‐Cessation"/

29. "Tobacco‐Use‐Disorder"/

30. Tobacco Smokeless.mp.

31. exp Tobacco Smoke Pollution/

32. exp Tobacco/

33. exp Nicotine/

34. exp Electronic Cigarettes/

35. ((quit$ or stop$ or ceas$ or giv$) adj5 smoking).ti,ab.

36. exp Smoking/pc, th [Prevention & Control, Therapy]

37. 27 or 28 or 29 or 30 or 31 or 32 or 33 or 34 or 35 or 36

38. 26 and 37
